# Quality Health Professions Programs: Path to Program of Merit Designation

**DOI:** 10.1093/geroni/igab046.2136

**Published:** 2021-12-17

**Authors:** Marilyn Gugliucci

**Affiliations:** University of New England College of Osteopathic Medicine, Kennebunk, Maine, United States

## Abstract

In 2015, the AGHE Program of Merit was expanded to implement a voluntary evaluation process for health professions programs that integrate gerontology/geriatrics competencies within their curriculum. These competencies augment students’ competence and confidence in the field of aging, preparing them to work with older adults and their care partners. All health professions and medical education programs are eligible to apply for the Program of Merit designation. This international process of evaluation: (1) Verifies program credibility and quality; (2) Informs campus administrators of global guidelines, expectations, and practice in aging for their health professions programs; (3) Assures quality graduates from POM designated programs; and (4) Clarifies for employers the knowledge and skills imparted to student graduates of POM designated health professions programs. Additionally, graduates from POM programs receive a certificate from AGHE stating the health profession program is a designated AGHE Program of Merit.

